# Response to letter regarding “*SCN9A* variant in a family of mixed breed dogs with congenital insensitivity to pain”

**DOI:** 10.1111/jvim.16707

**Published:** 2023-04-21

**Authors:** Rodrigo Gutierrez‐Quintana, Matthias Christen, Kiterie M. E. Faller, Julien Guevar, Vidhya Jagannathan, Tosso Leeb

**Affiliations:** ^1^ Small Animal Hospital, School of Biodiversity, One Health and Veterinary Medicine University of Glasgow Glasgow United Kingdom; ^2^ Institute of Genetics, Vetsuisse Faculty, University of Bern Bern Switzerland; ^3^ Royal (Dick) School of Veterinary Studies The University of Edinburgh Midlothian United Kingdom; ^4^ Department of Clinical Veterinary Sciences, Vetsuisse Faculty University of Bern Bern Switzerland

Dear Editor,

Thank you for the opportunity to respond to the letter from Drs. Taisuke Ishikawa and Hiroshi Aoki titled “Navigating the Pathogenicity of Candidate Gene Mutations: Spotlight on Paralog Nav Genes,” regarding our recent publication in JVIM titled “*SCN9A* variant in a family of mixed breed dogs with congenital insensitivity to pain.”^1^ We appreciate their interest in our publication and their useful and stimulating comments.

As mentioned by Drs. Taisuke Ishikawa and Hiroshi Aoki, in our publication we present several complementary lines of evidence that support the pathogenicity of the homozygous missense variant in *SCN9A*, XM_038584713.1:c.2761C>T or XP_038440641.1:(p.Arg921Cys) identified in our dogs. Unfortunately, we were unable to demonstrate its impact on the direct function of the Nav1.7 sodium channel, because we could not perform in vitro electrophysiological studies as previously reported.^2^, ^3^ We agree with Drs. Taisuke Ichikawa and Hiroshi Aoki that the presented data in our study is insufficient to conclusively prove the pathogenicity of the reported variant. For this reason, we reported the genetic variant as “likely” pathogenic or as “candidate” causative variant.

We completely agree that we should try to move toward functional annotation of mutated proteins in veterinary medicine and follow genetic guidelines used in humans, but doing so can sometimes be challenging. We believe there is still interest in reporting likely pathogenic variants of rare diseases promptly, to inform the veterinary community and avoid further breeding of potentially affected animals. For this reason, the use of functional findings in paralog variants in other species could be a very useful alternative. We did not consider this option in our publication, and we are very grateful to Drs. Taisuke Ishikawa and Hiroshi Aoki for their comment that provides further evidence of the pathogenicity of the reported variant in dogs with congenital insensitivity to pain.
